# Stability of Engineered Micro or Nanobubbles for Biomedical Applications

**DOI:** 10.3390/pharmaceutics12111089

**Published:** 2020-11-13

**Authors:** Beomjin Park, Semi Yoon, Yonghyun Choi, Jaehee Jang, Soomin Park, Jonghoon Choi

**Affiliations:** School of Integrative Engineering, Chung-Ang University, Seoul 06974, Korea; qjaqja0880@gmail.com (B.P.); semi103306@gmail.com (S.Y.); dydgus5057@gmail.com (Y.C.); jjaeh95@gmail.com (J.J.); soopark0801@gmail.com (S.P.)

**Keywords:** microbubble or nanobubble (MNB), stability, electrolyte, pH, stocking condition, ultrasonication

## Abstract

A micro/nanobubble (MNB) refers to a bubble structure sized in a micrometer or nanometer scale, in which the core is separated from the external environment and is normally made of gas. Recently, it has been confirmed that MNBs can be widely used in angiography, drug delivery, and treatment. Thus, MNBs are attracting attention as they are capable of constructing a new contrast agent or drug delivery system. Additionally, in order to effectively use an MNB, the method of securing its stability is also being studied. This review highlights the factors affecting the stability of an MNB and the stability of the MNB within the ultrasonic field. It also discusses the relationship between the stability of the bubble and its applicability in vivo.

## 1. Introduction

Micro/nanobubbles (MNBs) refer to structures that have a core separated from the periphery and is composed of gas. They are sized at the micro [1] or nano level [2]. The shell of an MNB distinguishes the external environment and the core that includes gases or drugs therein [3,4,5]. These bubbles not only serve as transporters that deliver gases or drugs into the body [6] but also act as contrast agents by contrasting vibration patterns of surrounding tissues with blood vessels by ultrasound [7].

Studies on the use of MNB for doxorubicin and gemcitabine delivery and transfection have been reported [8,9,10]. Advanced solid tumors, particularly, are in a hypoxic state compared to normal tissues owing to their characteristics of high angiogenesis, intercellular pressure, and frequent cell division, which are factors that prevent the action of drugs on tumors [11]. Nanobubbles are small and can penetrate solid tumors, which allows the infiltrating bubble to damage the surrounding tumor tissue and trigger an immune response due to the bubble itself being destroyed or the mechanism of releasing the drug and gas within. It is also possible to target a specific location in the body by changing the composition of the surface material or contents by giving the bubble an appropriate half-life or by binding functional groups [12,13]. MNBs are also used as ultrasound contrast agents. Unlike computed tomography (CT) and magnetic resonance (MR) imaging agents, MNBs are slow to be absorbed into the surrounding tissues and therefore remain in the blood vessels for a long time, facilitating the measurement of blood flow [14]. In particular, when diagnosing liver lesions, CT/MRI contrast agents diffuse into hepatic tissues, while ultrasound contrast agents, such as Sonovue, exist and act only in the arteries. This allows continuous real-time blood vessel images to be obtained. Additionally, when used as a contrast agent, the amount of gas in the core is an important factor in confirming the stability of the bubble. When evaluating the ultrasonic activity in several studies, the volume of bubble gas was identified as the main factor and determined the half-life. Even if the same amount of gas is delivered, nanobubbles are less affected by changes in volume concentration than microbubbles [15]. Cavitation, which is one of the characteristics of bubbles in an ultrasonic field, allows them to be used as an ultrasonic contrast medium. Changing several elements of the bubble can affect the aspect of cavitation, depending on the purpose [16]. For example, when applying sonothrombolysis, a technology that dissolves blood clots in blood vessels by causing cavitation using ultrasound, the use of MNB can lower the ultrasonic energy for cavitation. This reduces the burden on the patient when dissolving blood clots [17] and internal drugs can be released using cavitation [18]. Additionally, the combination of ultrasound (US) and bubble can increase drug permeability to BBB, with delivering antisense to BBB using US and bubble as an example [19]. Thus, there is a possibility that it can be used to diagnose and treat various brain diseases, such as brain tumors [20]. It is important to secure high bubble stability to leverage the above-described advantages of MNBs. For example, high stability in vitro allows the contents of the bubble to remain constant for a long period of time, while high stability in vivo allows drug release to be sustained for a longer period of time. The high stability in the ultrasound field is the basis for obtaining ultrasound images for a longer period of time. Generally, bubble stability in the body tends to be lower than bubble stability in vitro [21]. Securing high stability of the bubble not only increases the ease of storage but also allows for a longer clearer ultrasound image when used as a contrast agent, and also serves as the basis for long-term drug release from the body when used as a drug transporter.

**Figure 1:**
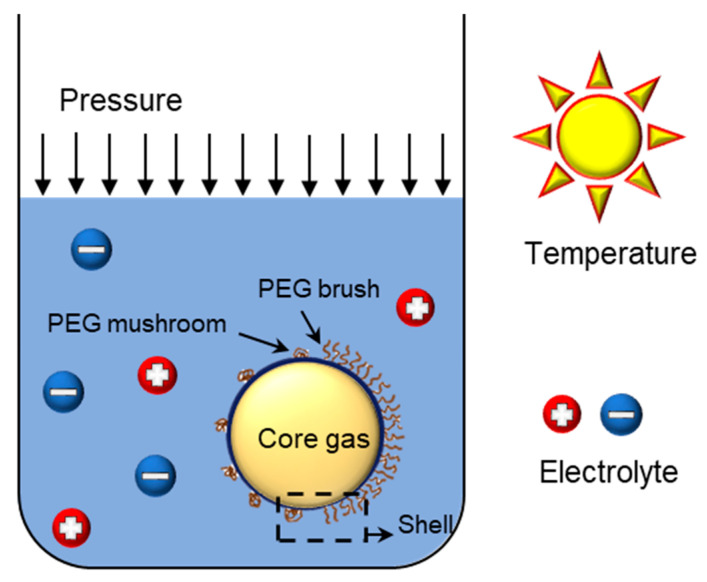
Factors affecting bubble stability.

The stability of these bubbles is determined by various factors, such as the composition of the shell and the type of central gas (Figure 1). In this review article, the factors that affect the stability of bubbles are first introduced, followed by a brief description of how to increase their stability. The relationship between the cavitation and stability of bubbles occurring in the ultrasonic field is also explained. Finally, the stability in vivo and techniques to increase stability by introducing polyethylene glycol (PEG) are discussed.

## 2. Bubble Structure

### 2.1. Shell

The shell structure of the bubble is not an essential element in the formation of bubbles [3,22]. However, the lifetime and the ultrasonic reverberation of a bubble with a shell is significantly higher than that of a bubble without an envelope [23]. In particular, the shell structure is essential to exhibit its performance as a contrast agent [24]. The outer shell of the bubble protects the inner material from the outside. It also applies pressure in the form of surface tension to prevent the material from spreading around. At the same time, the shell blocks direct contact of the bubble gas core with the external solvent. Furthermore, the bubble can be used as a contrast agent as the outer shell generates an ultrasonic echo that is distinct from the surrounding tissues. In theory, the life of a bubble is a few seconds [25]. However, bubbles with a lifespan of up to several months have been reported with a shell made of surfactants or similar substances [26]. Proteins, lipids, and polymers can also be used as the outer shell of the bubble [21,27,28,29]. When using lipids, amphiphilic substances, such as phospholipids, are used because they are self-assembled in a solvent and easily formed when creating bubbles [30]. Lipids mainly use phosphocholine (PC) and phosphatidylethanolamine (PE), the main components of the cell membrane, to reduce the possibility of phase change when bubbles are in physiological or storage conditions [31].

The stability of MNB varies depending on the chain length of the lipid and the degree of saturation/unsaturation. As the chain length becomes longer, the shape of the bubble becomes less spherical (therefore, shorter chains lead to more spherical shapes). Additionally, the bubble’s behavior varies according to the saturation/unsaturation state. Saturated lipids are more stable than unsaturated lipids. So, when used in ultrasound imaging, 1,2-distearoyl-sn-glycero-3-phosphocholine (DSPC), a long-chain saturated lipid, is often used [32,33]. For lipids that are used as the outer shell of the bubble, ultrasonic contrast agents, such as SonoVue^®^, are used. For proteins, bovine serum albumin (BSA) is used. For polymers, PEG and poloxamer can be used [34,35,36]. The function of the bubble can be changed by controlling the physicochemical properties of the shell material [37,38].

The outer shell of the bubble applies pressure in the form of surface tension to the center and affects the stability of the bubble [39]. As the radius of the bubble decreases, the pressure difference within and outside the bubble increases. Thus, the surface tension also increases, which then decreases the stability of the bubble. The issue of high surface tension of the shell can be solved using suitable additives, such as surfactants in the shell. It is notable that the life of air bubbles in water is very short [40], while nanobubbles that are naturally generated in the sea have a lifespan of 20 h or more. Thus, it has been reported that natural surfactants in the sea have the potential to increase bubble stability [41].

### 2.2. Core Gas

Bubble stability is affected not only by the shell but also by the core gas. The central gas affects the stability of the bubble because of its interaction with the surrounding solvent [42]. If several types of gases need to be used in the center of the bubble, selecting a gas with a low partition coefficient with the solvent to be stored is crucial for the stability of the bubble. Fluorine compound gas is a biologically inert gas that is safe for the human body. Additionally, it has low solubility in water and high solubility in oxygen [43]. Thus, it is widely used in oxygen supply through intravenous injection [44], for drug delivery [45], and in various medical studies [46]. Additionally, as fluorine gas has low bioactivity, it helps secure the biocompatibility of the MNB created using fluorine compound [47].

Bubbles whose centers are made of gas exert pressure in all directions through diffusion, while the periphery applies pressure on the gas. At this time, the pressure difference between the gas and the surrounding area changes the interface between the two into a curved surface, as the higher pressure pushes the interface. The interface changes into a curved surface, increasing the free energy to a level higher than when it was a flat surface, and pressure is applied to the gas in the form of surface tension. When the forces applied to both sides of the interface are in equilibrium, the bubble is stable. The pressure difference between both sides is expressed by the Young–Laplace equation [48] given by:

(1) $$P-P_{0}=2\gamma /R$$

where R is the radius of the bubble, γ is the surface tension, P is the pressure applied by the surroundings outside the interface, and P_0_ is the pressure applied by the center inside the interface. If there is a change in surface tension, radius, or pressure due to changes applied to the system, the state of the bubble changes, and a new stable state is established. When a change in one element is not balanced by a change in another, the bubble collapses.

In one study, F-hexane, F-triglyme, and F-diglyme were used as central gases, and the stability of the bubbles coated with DMPC was measured [47]. As a result of measuring the stability of the bubble using attenuation coefficient analysis, the sizes of the bubbles using the three gases were found to remain almost constant over time. However, the attenuation coefficient of the bubble using F-triglyme was found to decrease faster than those using the other two gases. The size, however, remained constant due to the merging of the bubbles. Although the number density of the bubbles decreased, the size remained constant, which is likely due to merging between bubbles. Additionally, in the experiment observing the total volume of bubbles over time, the bubbles using the above three gases tended to decrease the total volume slower than the bubbles using only air. The decrease in volume was slighter in the order of F-triglyme, F-diglyme, and F-hexane.

## 3. Stocking Condition

### 3.1. Zeta Potential, Electrolyte

If the surface tension of a bubble shell is high, the bubble is easily destroyed, even with a weak impact. Therefore, a surfactant is added to lower the surface tension of the bubble. It has been found that the addition of an electrolyte can replace the addition of a surfactant, as it lowers the critical micelle concentration of the surfactant [49].

The addition of the electrolyte affects the zeta potential of the bubble. If a buffer containing an electrolyte or salt is used, it is possible to stabilize complexes, such as liposomes, and control the surface charge by the electrolyte. The presence of an electrolyte, such as NaCl, in a nonionic solution enables surface charge regulation (SCR) of complexes, such as liposomes. For example, in the case of synthesizing a cationic liposome to deliver pDNA, a stable cationic lipoplex can be obtained by adding an appropriate amount of NaCl. This is through accelerated membrane fusion [50]. However, since cationic lipoplex easily interacts with surrounding cells and may result in cytotoxicity, it is advantageous to synthesize anionic liposome. In a later study, an anionic pDNA was mixed with protamine to make it cationic, and then a pDNA delivery system was developed using anionic liposome [51]. As suggested in the above study, bubbles can be stably synthesized by adding an appropriate amount of salt.

The ionized solvent or electrolyte, adsorbed around the charged colloidal particles, forms an electric double layer (EDL). The addition of the electrolyte changes the zeta potential and the thickness of the EDL, which affects the stability of the nanobubbles. The surface phenomenon controls most of the behaviors of the bubbles in the colloidal form. Bubbles with a neutral surface charge are difficult to maintain in a stable colloidal state and easily aggregated and lost. Bubbles with surface charges are stabilized by the electrostatic repulsion between bubbles. Zeta potential, which can indirectly estimate the surface charge, is used as an index for stability. The stability of colloidal particles varies depending on the type, but it is generally classified as stable when the absolute value of the zeta potential is 30 mV or more [52] (e.g., pH is one of the main factors of the zeta potential). As the acidity under storage conditions increases, the zeta potential increases in the positive direction from the existing value as the number of hydrogen ions in the solvent increases. By contrast, under basic conditions, the number of hydroxide ions tends to change in a negative direction [53]. It is difficult to explain the relationship between the concentration of the electrolyte and the zeta potential consistently. However, a low concentration electrolyte is adsorbed on the surface of the colloidal particles, increasing the zeta potential. Additionally, stability can be improved by forming an EDL to block the elution of the central gas. However, a high concentration of the electrolyte cancels the charge of the EDL and reduces bubble stability [54]. It has been highlighted that oxygen bubbles in an aqueous electrolyte solution have a longer lifespan than bubbles in pure water because ions form a thin layer around the bubbles [55]. MNBs without a shell tend to be negatively charged in water due to hydrated water molecules [56]. If an electrolyte is present, ions are collected around the surface of the charged bubble, and a thin film (EDL) is formed to reduce the elution of oxygen to the surrounding area. There is an effect that prevents the bubbles from merging owing to the presence of the same surface charge on the bubbles. However, excessive ions may cancel the surface charge and reduce the stability of the bubbles. When the amount of charge of the added electrolyte is high, even if the same number of ions gather around the bubble and block the elution of the central gas, the surface charge to be canceled increases, and thus the surface charge is greatly reduced [57]. As the surface charge approaches zero, the possibility of merging bubbles increases. Thus, an electrolyte containing monovalent ions is effective when adding an electrolyte for long-term storage.

### 3.2. Pressure, Temperature

For a bubble to exist in a state that maintains a certain shape, the pressure inside and outside the bubble must be balanced. The internal pressure is determined by the pressure exerted by the center toward the interface. The external pressure becomes the pressure applied by the solvent toward the interface. If the pressure difference with height is neglected during storage, this pressure is determined by the pressure exerted by the atmosphere on the solvent, i.e., atmospheric pressure. Increasing the pressure, in turn, decreases the size of the bubble and increases the solubility of the solvent in the gaseous center. This promotes the elution of the center [27].

When the outer shell is composed of lipids and is highly elastic, a wrinkling transition, described later in the paper, occurs such that a certain degree of resistance to the loss of the central gas may be obtained. On the contrary, if the outer shell is composed of protein, and the ambient pressure increases, buckling occurs and deforms the structure. At this time, the critical pressure is determined by the thickness, Young’s modulus of the bubble shell, and the diameter of the bubble [27].

A change in temperature also affects the life of the bubble through changes in internal and external pressure and solubility in the center of the solvent. When the temperature increases, the central gas expands according to the ideal gas law. As the temperature of the surrounding solution increases, the gas solubility in the solvent decreases, and the gaseous molecules flow back into the bubble, causing the bubble to expand. Thus, the elastic stress applied to the membrane increases, and the viscoelasticity of the stretched shell decreases [58]. Bubbles are stored freeze-dried, and the ones used as contrast agents are stored after being lyophilized and mixed with water in many cases before use. It has been reported that it has better storage stability when stored by lyophilization. Studies have shown that lyophilization can be stored for at least one year [59,60,61].

### 3.3. Initial Bubble Concentration

When storing bubbles, the initial concentration of the solvent affects the life of the bubbles. The central part inside the bubble tends to diffuse and escape owing to the difference in concentration with the surrounding area. Therefore, if the concentration of the solvent is higher than that of the core, the bubble remains stable. If the initial concentration of the bubble is high, the volume of the center eluted per bubble decreases to saturate the same volume of solvent, enabling the reduction of the change in the bubble. This is called the diffusive shielding effect [62].

## 4. Bubble Stability in the Ultrasonication Field

**Figure 2:**
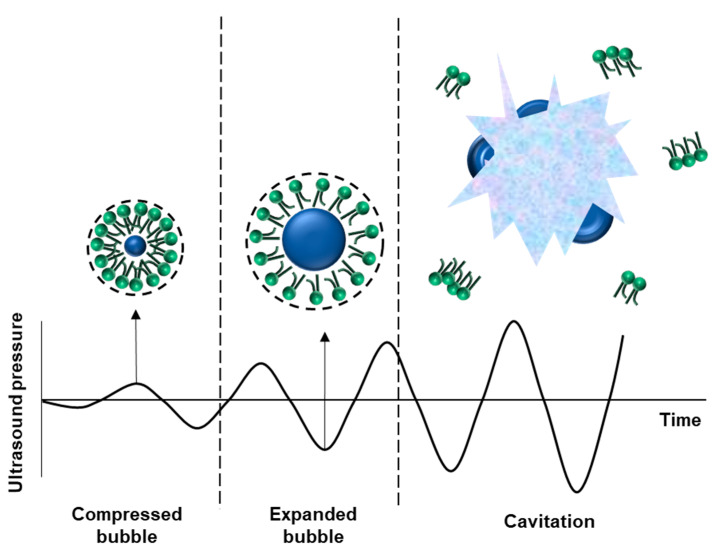
State of the nanobubble in the ultrasonic field. When the bubble enters the ultrasonic field, it oscillates. If frequency overcomes the threshold, cavitation occurs.

It has been found that bubbles vibrate when exposed to ultrasonic waves (Figure 2). Additionally, the vibration of the bubble appears differently based on the acoustic pressure applied. The ultrasonic device is designed with the ability to control the acoustic pressure by adjusting the mechanical index (MI). This is done by dividing the peak rare fractional pressure of the ultrasonic wave by the square root of the ultrasonic center frequency. Living tissues produce linear vibrations under an ultrasonic field. When the MI is low, the bubble causes a linear vibration with a similar degree of contraction and expansion. In a slightly higher MI, they cause a nonlinear vibration, where the microbubbles expand more than they contract. This nonlinear vibration represents a signal that is distinct from the linear vibration of the surrounding tissue [24]. When the MI exceeds a critical point, the bubble is destroyed, and for a short duration, sends a signal that is distinct from the surrounding tissues in the form of nonlinear vibration [63].

Moreover, a stable cavitation occurs when bubbles cause nonlinear vibration, while inertial cavitation occurs when the bubbles are destroyed. When cavitation occurs, a physical change in the surrounding cell structure takes place. Additionally, the gas inside the bubble is eluted, and a strong shock wave, shear force, and micro-jet are generated [64]. These changes include the transformation or degradation of vesicles that can cause changes in lipid cell membranes, even without inertial cavitation [65].

Additionally, some of the ultrasonic energy is converted into thermal energy and absorbed by the surrounding tissues and their temperature increases. The tissues that absorb heat energy actively exchange materials, thereby increasing the delivery efficiency of gases or drugs inside the bubble. When a bubble is exposed to ultrasonic waves to cause cavitation, the occurrence of stable or inertial cavitation is influenced by several factors, including the characteristics of the ultrasonic wave, the size and composition of the bubble, and the surrounding environment. Several models have been developed to predict the phenomenon of a single bubble. The Rayleigh–Plesset equation, which expresses the bubble behavior in an incompressible fluid, is often used to develop these. Bubbles in the ultrasonic field can cause cavitation.

Cavitation is affected by the surrounding environment, such as acoustic pressure, center frequency, pulse length, and characteristics of the medium. Acoustic pressure refers to the change in pressure generated by sound waves passing through a medium. Acoustic pressure takes the form of a sine wave in a time–pressure relationship, and there are peaks and lows. The lowest point of the acoustic pressure is called the peak-rarefactional pressure, and this is related to the fragmentation threshold since the bubble expands. Mainly, the fragmentation threshold means the minimum acoustic pressure for inertial cavitation to occur. In addition, when applying continuous ultrasound, the fragmentation threshold increases as the frequency increases [66]. When pulsed ultrasound is applied, the threshold decreases as the ultrasound application time increases. This is because bubbles stabilize during the off-time interval of pulsed ultrasound [67].

Compared to a bubble without a shell, a bubble with a shell is 20 times harder at the interface. Thus, the frequency of ultrasonic waves for cavitation is also higher. Additionally, the thicker the bubble shell, the higher the frequency of ultrasonic waves required for cavitation. The frequency of the ultrasonic wave required also varies according to the composition of the shell because the movement of the bubble shell is influenced by the surface tension and the volume viscosity coefficient between the bubble and the surrounding liquid. The higher the viscosity coefficient of the outer shell, the higher the energy required for vibration, and a higher frequency ultrasonic wave must be applied. Additionally, the image quality of the microbubbles is superior to that of the nanobubbles in the same ultrasonic wave because the nanobubbles are smaller and cause less vibration even when the same ultrasonic wave is applied. Research has shown that a larger volume of microbubbles amplifies an image signal better [68]. Thus, microbubbles are more advantageous than nanobubbles in increasing the permeability of the material emitted by the bubble into the blood vessel because the degree of vibration of microbubbles in the ultrasonic field is larger than that emitted by the nanobubbles [69].

When a bubble is cavitating under the influence of ultrasonic waves, a temporary hole is created in the surrounding cell membrane, allowing foreign substances to enter the cell. Thus, drugs can be delivered into the solid tumor through cavitation. A study shows that using focused ultrasound (FUS) limits the cavitation area of the bubble and increases the efficiency of drug delivery [50].

Recent studies have shown that cavitation alters the permeability of blood vessels and cell membranes through three mechanisms [70]:

1. The cell membrane potential changes favorably to ensure the inclusion effect, while there is a regular mechanical load exerted on the cell membrane by the vibration of the bubble by stable cavitation.
2. The volume of the vibrating bubble changes as it changes from its stable state to inertial cavitation. Accordingly, the gap between the vascular endothelial cells is temporarily increased, the cohesive force is weakened, the diffusion of reactants is strengthened, and absorption into the tissue is increased.
3. Temporary pores, based on ultrasonic treatment caused by inertial cavitation, are created in vascular endothelial cells, and the inclusion of large molecules in the cell increases.

## 5. In Vivo Application

Currently, bubble stability has been the most significant barrier to their application in vivo. This is because, as the environment inside the test tube and the organism differs, it is difficult to expect that the performance of the bubble in the test tube will be similar to that in vivo. According to one study, the stability of the bubble in vivo and in vitro showed the same tendency. However, the stability in vivo was lower. DEFINITY^®^, an ultrasonic contrast agent currently in use, has a lifetime of approximately 3 to 5 min [71].

There are two main reasons bubbles are destroyed in vivo:

1. Destruction by phagocytosis by the body’s immune system.
2. Central gas elution and destruction by physical impact.

Among these, bubble loss caused by the body’s immune system is mainly due to the phagocytic action of macrophages, liver, and spleen. Reducing the resulting loss is possible through the deformation of the bubble shell. PEGylation is an example that will be discussed later.

The elution of the central gas and destruction due to physical shock occurs when the central gas dissolves and escapes into the surrounding blood that the bubble is in contact with in vivo. Additionally, when bubbles exist in the blood vessels, they may be physically destroyed due to blood flow. However, it is difficult to reproduce the conditions in which the blood vessel bubbles are physically destroyed in vitro. In the report by Borden et al., wrinkling transition was mentioned as a method to ensure the stability of a lipid bubble in vivo [71]. When the central gas of the bubble escapes, and when the length of the acyl chain of the lipid is long (more than 18 carbons), the bubble does not show only a spherical shape, but a somewhat crushed shape and is maintained. However, when the length of the acyl chain is short (less than 16 carbons), the bubble retains only a spherical shape and decreases in size rapidly with the elution of the central gas. Therefore, it is advantageous to use a core gas that exhibits low solubility in the outer shell and surrounding environment, which can exhibit wrinkling transition to secure stability in vivo [71].

PEGylation is a widely used surface modification method to increase the duration [72] or stability [73] of various drugs. Particularly, as the method has various advantages, such as wide applicability and high biocompatibility, it is applied in various fields to increase drug delivery efficiency.

The PEGylation of a bubble changes its stability. When applied in vivo, PEGylation helps to avoid various side effects caused by the immune system, such as the reticuloendothelial system, to increase its half-life and facilitate targeting by binding various ligands. Additionally, applications, such as timely drug delivery, in connection with ultrasound are also possible. PEG on the bubble surface exists in two forms: mushroom and brush [74]. This morphology is affected by the mole fraction of the PEG and the density and length of the chain.

PEGylation enables the avoidance of the immune reaction as PEG blocks access to various proteins and enzymes around the bubble. It was observed that as the concentration of PEG on the bubble surface increased, the binding of avidin and the biotinylated membrane decreased [75]. Particularly, at a PEG concentration of 6 mol% or more, PEG was present in the mushroom form, and there was only a little binding of avidin. This binding resistance is due to the repulsive force produced by the PEG. For avidin to bind with biotin, the PEG present on the membrane must be pushed aside, and sufficient binding sites must be secured. This process requires additional energy consumption. Therefore, it blocks the access of other substances to the bubble surface. It was confirmed that it had a similar effect on lipids by blocking the fusion of excess lipids to the existing bubbles. However, once the fused lipid was dissociated, it showed a pattern that was independent of the PEG concentration.

Additionally, as the concentration of PEG increases, the range and size of the repulsive force increase accordingly. At low pressure, the interlayer distance of the bubble tended to increase to 8, 12, and 13.4 nm when the concentration of PEG2000 was 1.5%, 5%, and 10%, respectively. PEG in the form of a brush can be used as a spacer to position the antibody beyond the PEG mushroom. This spacer binds to the hydrophilic site of the phospholipid, and is located in the distal part of the bubble and can bind to other receptors [76]. Combining these effects well can impart both immune evasion and target specificity to the bubble.

## 6. Summary

This review article briefly introduces the factors that affect the stability of MNBs. MNBs are attracting attention as drug transporters with high biocompatibility, flexibility according to the purpose of use, and versatility in application. Particularly, MNBs are used as contrast agents for ultrasound imaging and have already been commercialized. They can be used not only for the delivery of drugs but also for the delivery of oxygen or genetic material. If the characteristics of MNBs can be maintained constant for a long duration, the applicable range of MNBs will expand significantly and aid the development of diagnosis and treatment technology.
